# Risk Assessment in Cannulation for Minimally Invasive Heart Valve Surgery: The Modified HOSTILE Score

**DOI:** 10.3390/jcm15020843

**Published:** 2026-01-20

**Authors:** Jacqueline Kruse, Miriam Silaschi, Michael Celik, Marwan Hamiko, Eissa Alaj, Hossien Alirezaei, Atsushi Sugiura, Enzo Lüsebrink, Sebastian Zimmer, Farhad Bakhtiary

**Affiliations:** 1 Department of Cardiac Surgery, University Hospital Bonn, 53127 Bonn, Germany; miriam.silaschi@ukbonn.de (M.S.); michael_celik@web.de (M.C.); marwan.hamiko@ukbonn.de (M.H.); eissa.alaj@ukbonn.de (E.A.); hossien.alirezaei@ukbonn.de (H.A.); farhad.bakhtiary@ukbonn.de (F.B.); 2 Department of Medicine II, Heart Center Bonn, University Hospital Bonn, 53127 Bonn, Germany; atsushi.sugiura@ukbonn.de (A.S.); enzo.luesebrink@ukbonn.de (E.L.); sebastian.zimmer@ukbonn.de (S.Z.)

**Keywords:** minimally invasive heart valve surgery, CT imaging, risk assessment

## Abstract

**Objectives:** The HOSTILE score was developed to assess femoral access challenges in transcatheter valve therapy. Similar vascular issues arise in femoral cannulation for minimally invasive valve surgery, making CT-based planning essential. We adapted the score for surgical use (MOD-HOSTILE) and evaluated its association with neurological and adverse outcomes. **Methods:** In this single-center retrospective study, the MOD-HOSTILE score (0–11 points) was calculated for 364 patients undergoing minimally invasive heart valve surgery from 2019 to 2023. Patients were stratified into low (0–2), mild (>2–5), and high (>5–11) score categories. Outcomes included 30-day stroke, other neurological events, and perioperative complications. **Results:** High MOD-HOSTILE patients were significantly older (70 [64.7–73.0] vs. 61 [60.0–63.0] years; *p* < 0.01) and had higher surgical risk (EuroSCORE II 1.79 [1.26–2.16] vs. 0.83 [0.75–0.94]; *p* < 0.01). Neurological complications were more frequent in the high MOD-HOSTILE group, including stroke (8.7% vs. 0.9%; *p* = 0.02) and hemiplegia (13.0% vs. 0.9%; *p* < 0.01). Axillary cannulated patients had higher MOD-HOSTILE scores than femoral cannulated patients. Stroke risk was highest in patients with high MOD-HOSTILE score undergoing axillary cannulation (high vs. low MOD-HOSTILE, 18.2% vs. 0%; *p* = 0.04). Thirty-day mortality was comparable between groups (*p* = 0.09). MOD-HOSTILE predicted stroke with an AUC of 0.78 (95% CI 0.73–0.82) and OR 1.4 per point (95% CI 1.1–2.0). **Conclusions:** The MOD-HOSTILE score identifies vascular and neurological risk in minimally invasive valve surgery, with scores ≥5 indicating elevated risk of stroke and delirium. Patients with high scores may benefit from alternative surgical strategies.

## 1. Introduction

In transcatheter aortic valve replacement (TAVR), transfemoral access is the preferred route of valve delivery. The HOSTILE score was developed to aid assessment of suitability for transfemoral access and to identify patients with “hostile” iliofemoral anatomy, characterized by small vessel diameter, severe calcification, and significant vascular tortuosity, which are known to increase the risk of access-related complications during TAVR [1,2,3].

In contrast to TAVR, minimally invasive valve surgery requires prolonged large-bore arterial cannulation (typically 16–19 Fr) to establish cardiopulmonary bypass, resulting in sustained retrograde arterial perfusion. This exposes patients to continuous hemodynamic shear stress, prolonged embolic exposure, and altered cerebral perfusion dynamics, all of which have been associated with neurological complications in the setting of peripheral cannulation and are not adequately captured by the original HOSTILE score [4,5]. Femoral arterial cannulation represents the most commonly used access route in minimally invasive cardiac surgery because of its technical simplicity, widespread familiarity, and feasibility for percutaneous cannulation, but concerns persist regarding retrograde embolization in patients with atherosclerotic disease of the aorta or iliofemoral vessels [6,7,8].

We, therefore, modified the existing HOSTILE score (MOD-HOSTILE) to support preoperative decision-making in endoscopic minimally invasive valve surgery, where femoral vessels are routinely cannulated for extracorporeal circulation. In this surgical setting, vascular access is not only a determinant of technical feasibility but also a critical factor influencing retrograde perfusion, embolic load, and neurological risk [4,9,10]. To reduce the potential risks associated with retrograde perfusion, axillary arterial cannulation providing antegrade flow has been adopted as an alternative strategy in selected patients with extensive aortic or peripheral vascular disease identified on preoperative computed tomography [4,5,9,10].

Several centers have proposed CT-based algorithms to guide the choice between femoral and axillary cannulation, using criteria such as thrombus thickness, circumferential calcification, and severe iliofemoral tortuosity; however, these strategies are largely derived from retrospective analyses and heterogeneous cohorts [4,5,10].

While meta-analyses and registry studies from aortic surgery populations suggest that axillary cannulation may reduce short-term mortality and neurological complications in high-risk patients, evidence specific to minimally invasive valve surgery remains limited and inconsistent, and randomized data are currently lacking [2,5,10].

The MOD-HOSTILE score was developed to better reflect the cumulative vascular disease burden relevant to minimally invasive valve surgery, incorporating anatomical and calcific features that may predispose patients to access-related and neurological complications due to peripheral cannulation. We hypothesized that higher MOD-HOSTILE values are associated with an increased risk of stroke and neurological events in minimally invasive valve surgery.

**Figure 1:**
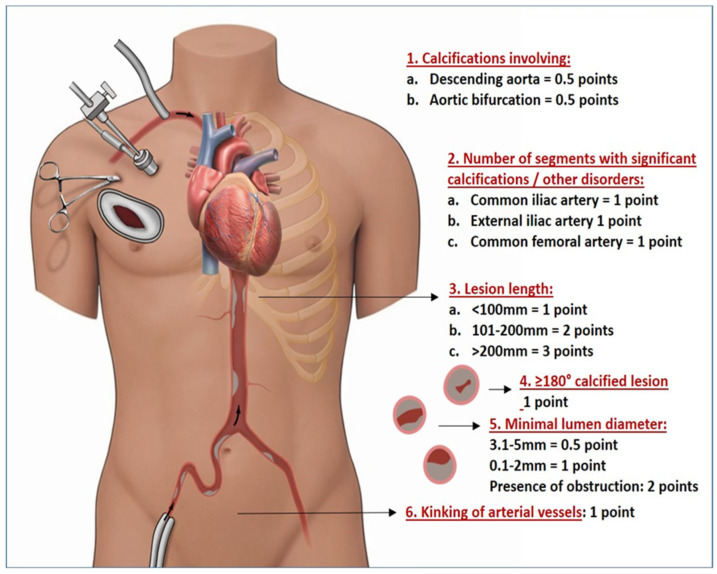
Operative set-up for minimally invasive valve surgery, including MOD-HOSTILE criteria and the amount of points for each pathological criteria.

The present study evaluates the predictive value of the MOD-HOSTILE score and explores its potential role in guiding vascular access strategy in this patient population (Figure 1).

By integrating vascular risk stratification with preoperative imaging, the MOD-HOSTILE score may help identify patients who could benefit from alternative cannulation strategies and more individualized surgical planning in minimally invasive valve surgery [4,5,9,10].

## 2. Materials and Methods

This single-center, retrospective study included all patients who underwent minimally invasive, endoscopic heart valve surgery at our institution between 2019 and 2023 and had baseline ECG-gated contrast-enhanced CT scans from neck to groin (n = 364). Procedures were performed via a right anterolateral minithoracotomy (RAMT) and comprised isolated aortic, mitral, or tricuspid valve surgery as well as combined valve procedures.

Patients with active infectious endocarditis were excluded from the analysis. Demographic, preoperative, intraoperative, and postoperative data were collected from the institutional database, and the MOD-HOSTILE score was calculated for each patient to assess vascular risk. Also, patients with ascending aortic disease and arch disease were not treated via minimally invasive access at that time at our center, as this was defined to be a contraindication; therefore, it is not part of the MOD-HOSTILE score.

Arterial cannulation was performed mainly via the femoral or axillary artery using standard peripheral cannulation techniques.

At our center, patients scheduled for minimally invasive cardiac surgery with peripheral cannulation routinely undergo preoperative CT angiography of the supra-aortic and iliofemoral vessels, minimizing the risk of spectrum bias.

**Figure 2:**
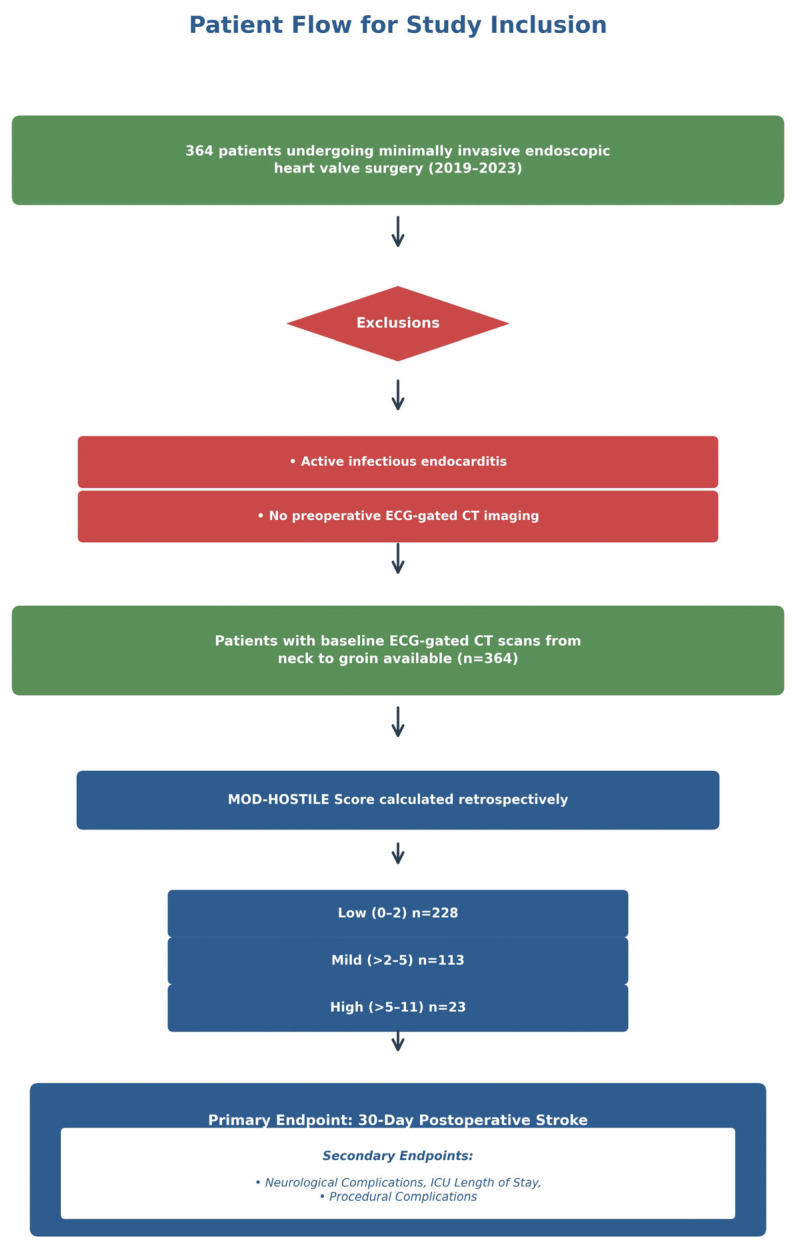
Flowchart of patient selection and MOD-HOSTILE score assessment for minimally invasive endoscopic heart valve surgery.

The flow of patient selection, including inclusion and exclusion criteria, availability of ECG-gated CT scans, and retrospective calculation of the MOD-HOSTILE score, as well as the assessment of primary and secondary endpoints, is illustrated in Figure 2.

The original HOSTILE score was initially developed for transcatheter aortic valve replacement (TAVR) to obtain vascular access challenges based on anatomical criteria. It includes CT-derived features such as the number of iliofemoral segments with significant lesions, a total lesion length greater than 100 mm, involvement of the aortic bifurcation, the presence of lesions in tortuous vessel segments, circumferential calcification exceeding 180°, a minimal luminal diameter < 5 mm, and vessel obstruction. These factors were combined to predict the risk of vascular complications, particularly in patients with peripheral arterial disease undergoing TAVR [5].

The MOD-HOSTILE score represents a clinically motivated re-specification of the original HOSTILE score, adapted to support pre-operative decision-making in endoscopic minimally invasive valve surgery. The score is calculated based on the following factors:

- Calcifications in the descending aorta and/or aortic bifurcation;
- Number of segments with significant calcifications (including the common iliac artery, external iliac artery, and common femoral artery);
- Length of vessel calcification or lesions;
- >180° calcified lesion;
- Minimal luminal diameter of the vessel;
- Kinking of arterial vessels.

The MOD-HOSTILE score ranges from 0 to 11 points (Figure 1). We assessed the score in 364 patients undergoing minimally invasive valve surgery (MIVS) with available ECG-gated CT scans (2019–2023) and categorized them into three groups: low (0–2), mild (>2–5), and high (>5–11) anatomical adversity. We defined significant calcification according to dense plaque with high Hounsfield Units (typically > 130 HU on non-contrast CT) or a calcium thickness of >2 mm, and we also gave a point when the calcification in the common femoral artery was located on the anterior wall at the level of the puncture site. Vessel kinking was defined by whether several pronounced curves/bends or an angle >90° between segments occurred. Femoral cannulation was performed percutaneously using closure devices, while axillary access was achieved via surgical cut-down with direct cannulation without grafts. The cannulation strategy was decided upon pre-operative CT scans. The MOD-HOSTILE score in this study was analyzed retrospectively and not pre-operatively.

The study’s primary endpoint was the occurrence of stroke within 30 days after surgery. Stroke is defined according to AHA/ASA criteria as a new focal neurological deficit lasting more than 24 h or an imaging-confirmed acute ischemic or hemorrhagic infarction. Transient ischemic attack (TIA) was defined as a transient episode of neurological dysfunction caused by focal brain, spinal cord, or retinal ischemia, without evidence of acute infarction on neuroimaging. Temporary neurological complications were defined as short-term neurological symptoms arising peri- or postoperatively that are less pronounced than a TIA and not necessarily of central origin (e.g., numbness, tingling, or transient visual disturbances).

Secondary endpoints included a broader set of neurological complications, such as monoplegia, paraplegia, hemiplegia, seizures, coma, aphasia, and intraoperative drops in near-infrared spectroscopy (NIRS) of more than 20%, as well as complications related to the vascular access site. Seizure was defined as a transient occurrence of signs and/or symptoms resulting from abnormal, excessive, or synchronous neuronal activity, clinically diagnosed based on witness reports, EEG findings, or medical documentation. Coma was defined as a state of sustained unresponsiveness despite the absence of sedative medication, in which the patient cannot be aroused, fails to respond purposefully to external stimuli, and exhibits no eye opening, persisting for at least one hour, with a Glasgow Coma Scale (GCS) score ≤ 8. Aphasia was defined as an acquired language disorder characterized by impaired ability to produce and/or comprehend spoken or written language, as determined by clinical examination or validated language assessments.

All endpoints were assessed up until hospital discharge, and all outcomes represent in-hospital events without further follow-up. The MOD-HOSTILE score was analyzed as a continuous variable for all inferential analyses. An exploratory threshold analysis using a cut-off of ≥5 was performed based on observed stroke rates (8.7% vs. 0.88%). When presented descriptively, score categories are applied consistently, with exact cut-offs provided in table footnotes or figure legends. Secondary outcomes also included the composite neurological endpoint and ICU length of stay, alongside other procedural and clinical endpoints, ensuring consistency throughout the manuscript.

Postoperative strokes were confirmed using cranial computed tomography (cCT) and CT angiography, allowing precise evaluation of both brain parenchyma and cerebral vasculature. The equipment was sourced from Philips Healthcare (Best, The Netherlands).

### 2.1. Treatment of Neurological Complications

Postoperative management across the MOD-HOSTILE groups included anticoagulation, antiplatelet therapy, physiotherapy, neurological rehabilitation, and, when indicated, neurointerventional procedures such as thrombectomy or lysis therapy. These interventions were applied according to standard institutional protocols in golden time.

### 2.2. Statistical Analysis

Categorical variables are presented as absolute counts and percentages. Continuous variables were tested for normality using Shapiro–Wilk test within each MOD-HOSTILE score category. Normally distributed variables are reported as mean ± standard deviation, whereas non-normally distributed variables are presented as median with interquartile range (25th–75th percentile).

Comparisons across MOD-HOSTILE score categories (low, mild, and high) were performed using one-way analysis of variance (ANOVA) for normally distributed variables and the Kruskal–Wallis test for non-normally distributed variables. A single global *p*-value is reported for each variable to indicate overall differences between groups.

Logistic regression and receiver operating characteristic (ROC) analyses were performed to assess the predictive performance of the MOD-HOSTILE score for postoperative stroke. Model discrimination and calibration were evaluated using ROC analysis and calibration plots, respectively. The cannulation route was not included in the multivariable logistic regression model to avoid multicollinearity since the cannulation route was highly correlated with higher MOD-HOSTILE categories.

Decision curve analysis (DCA) was performed to evaluate the clinical usefulness of the MOD-HOSTILE score by quantifying the net benefit across a range of clinically relevant threshold probabilities.

All statistical analyses were conducted using MedCalc Version 23.1.7—32-bit (MedCalc Software Ltd., Ostend, Belgium). Figures were generated using GraphPad Prism (version 10.4.1). Decision curve analysis was performed using R (R Foundation for Statistical Computing, Version 4.5.2; Vienna, Austria).

## 3. Results

### 3.1. Overall Cohort

From the overall cohort, 47.83% (11/23) were cannulated axillary and 52.17% (12/23) femoral in the high MOD-HOSTILE group. Patients with higher MOD-HOSTILE scores experienced more comorbidities and adverse events, consistent with the observed associations between vascular risk and perioperative complications.

Patients with a high MOD-HOSTILE score were older (median 70.00 [64.70–73.00] years) compared to the low score group (61.00 [60.00–63.00]; *p* < 0.01). They had higher EuroSCORE II (1.79 [1.26–2.16] vs. 0.83 [0.75–0.94]; *p* < 0.01) and tended to have lower renal function (eGFR 75.57 [68.36–84.34] vs. 88.17 [83.63–90.00]; *p* < 0.01). Prevalence of COPD (17.39% vs. 3.07%; *p* < 0.01) and history of smoking (56.52% vs. 16.23%; *p* < 0.01) were higher in the high score group. BMI, BSA, atrial fibrillation, history of stroke, and diabetes mellitus did not differ significantly (see Table 1).

Intraoperative characteristics differed between groups. Aortic valve replacement was more frequent in high MOD-HOSTILE patients (43.48% vs. 20.61%; *p* < 0.01), whereas mitral valve repair/replacement was more common in the low score group (72.37% vs. 47.83%; *p* < 0.01). Axillary cannulation was more often performed in high score patients (47.83% vs. 7.02%; *p* < 0.01), while femoral cannulation predominated in low score patients (92.98% vs. 52.17%; *p* < 0.01). Cross-clamp time and intraoperative NIRS drop ≥20% during CPB were similar across groups.

**Figure 3:**
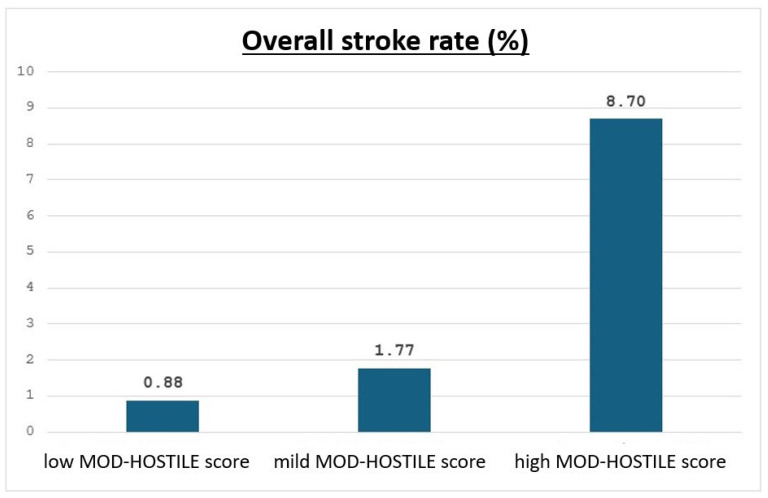
Overall stroke rate for MOD-HOSTILE categories (*p* = 0.03).

**Figure 4:**
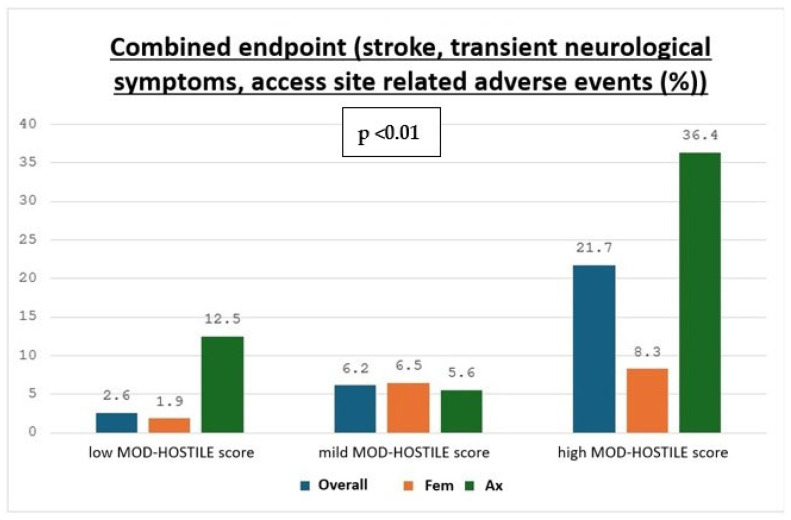
Combined Endpoint including stroke, transient neurological symptoms, and access site-related adverse events for MOD-HOSTILE categories stratified by cannulation route and overall.

Postoperative neurological events were more frequent in high MOD-HOSTILE patients. Stroke occurred in 8.70% of high score patients vs. 0.88% in the low group (*p* = 0.02, see Figure 3), hemiplegia in 13.04% vs. 0.88% (*p* < 0.01), and temporary neurological complications in 8.67% vs. 1.75% (*p* = 0.01). The combined neurological endpoint occurred in 21.74% of high score patients compared to 10.69% in the low group (*p* = 0.12). Delirium, seizures, TIA, and ICU stay were comparable between groups (see Figure 4).

Blood product use was higher in high MOD-HOSTILE patients: pRBCs median 2.00 [0.00–2.65] vs. 0.00 [0.00–0.00] (*p* = 0.01). Hospital stay was slightly longer in high score patients (11.00 [7.00–13.65] vs. 10.00 [9.00–10.00]; *p* < 0.01).

**Figure 5:**
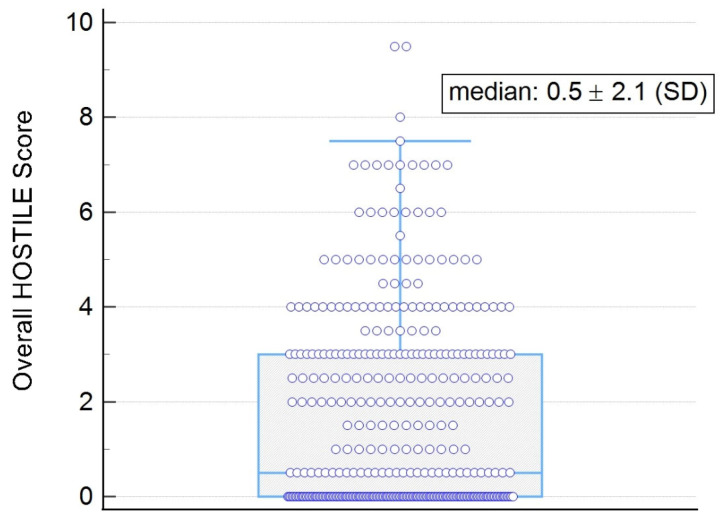
Overall result of the MOD-HOSTILE score.

Despite these differences, 30-day mortality did not differ significantly between groups (0% in high vs. 1.32% in low; *p* = 0.09). The median MOD-HOSTILE score for the overall cohort is shown, with individual values represented as points in Figure 5.

#### 3.1.1. ROC Curve

**Figure 6:**
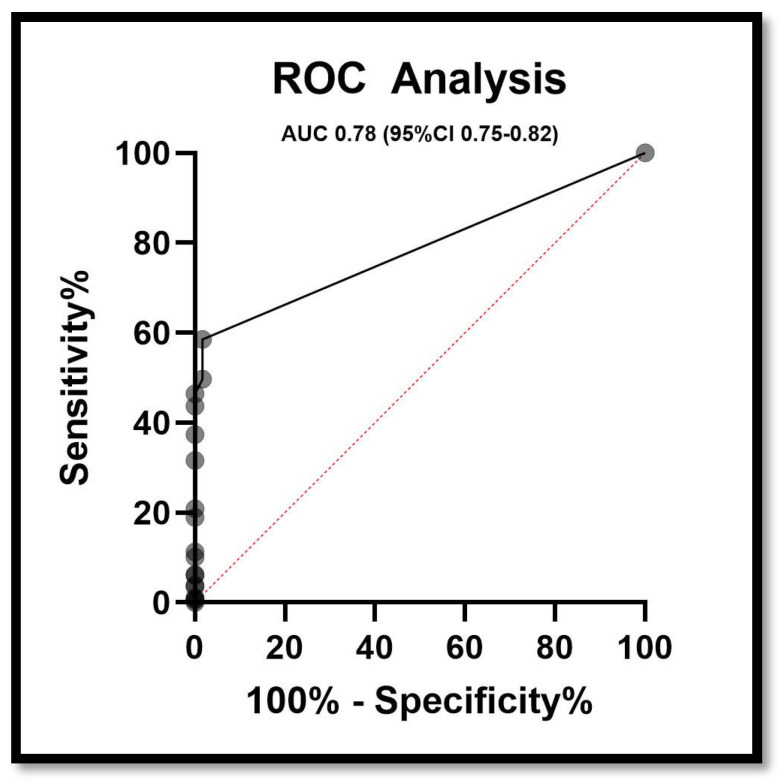
Receiver operating characteristic (ROC) curve of the MOD-HOSTILE score for the prediction of postoperative stroke. The area under the curve (AUC) was 0.78 (95% CI 0.75–0.82), indicating good discriminatory ability.

The receiver operating characteristic (ROC) curve demonstrates the predictive performance of the MOD-HOSTILE score for postoperative stroke. The model showed good discriminatory ability, with an area under the curve (AUC) of 0.78 (95% confidence interval, CI 0.75–0.82) (Figure 6).

#### 3.1.2. Calibration Curve

**Figure 7:**
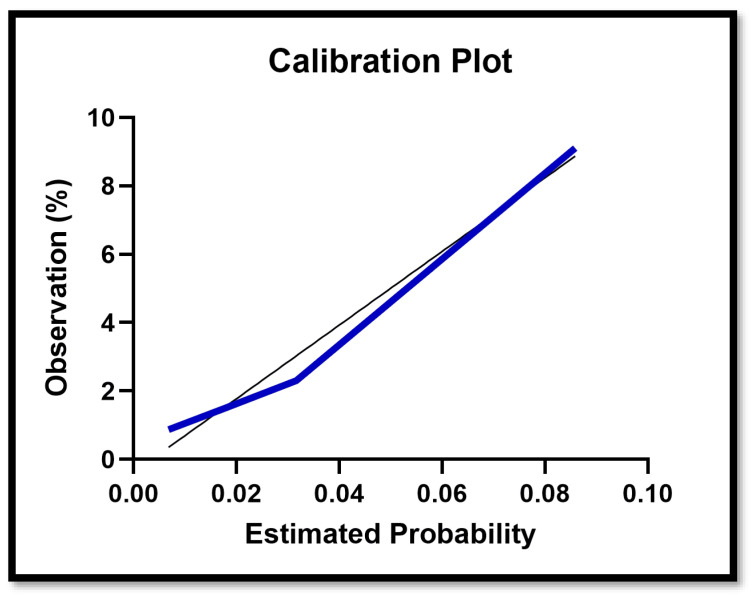
Calibration curve showing agreement between predicted and observed stroke probabilities for the MOD-HOSTILE score.

The calibration plot shows good agreement between predicted and observed stroke incidence across the range of predicted risks, indicating adequate calibration of the logistic regression model (Figure 7).

#### 3.1.3. Multivariate Logistic Regression

In multivariate logistic regression including age, peripheral artery disease, extracranial carotid stenosis >50%, and MOD-HOSTILE score categories, only a high MOD-HOSTILE score was significantly associated with postoperative stroke (odds ratio [OR] 11.63, *p* = 0.03). Other variables, including age, peripheral arterial disease, and extracranial carotid stenosis >50%, were not statistically significant.

#### 3.1.4. Comparison with Combined Neurological Endpoint

A secondary analysis using a combined endpoint of neurological complications (stroke and other events) included more events (50 vs. 6 for stroke alone) and produced a statistically significant overall model (*p* < 0.01). However, the discriminatory ability of this model was lower (AUC = 0.66), and the MOD-HOSTILE score was not the strongest predictor. This supports focusing on stroke as the primary outcome, where the score shows its clearest predictive value.

#### 3.1.5. DCA (Decision Curve Analysis) Curve

Decision curve analysis demonstrated that the MOD-HOSTILE score provided a higher net benefit than the treat-none strategy at lower threshold probabilities, while remaining below the treat-all strategy across the entire range of evaluated thresholds. The first intersection between the MOD-HOSTILE curve and the treat-none line occurred at a threshold probability of approximately 5%. Below this threshold, the MOD-HOSTILE curve remained above the treat-none strategy, indicating a positive net benefit within this range.

**Figure 8:**
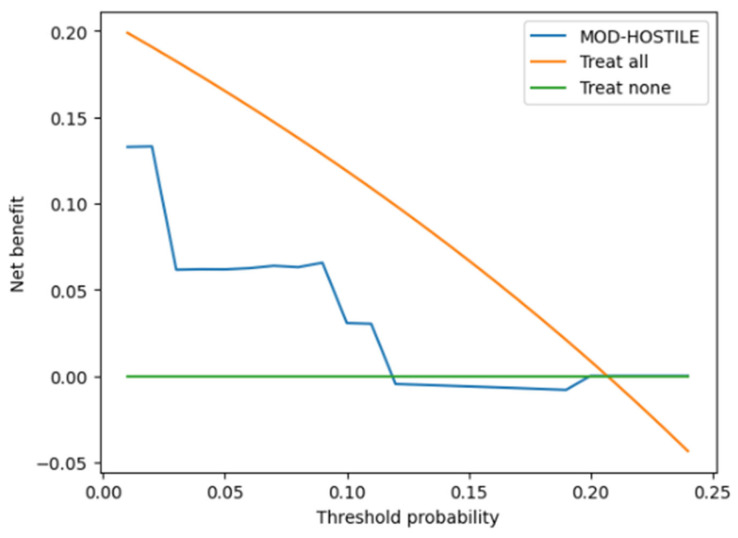
Decision curve analysis for prediction of 30-day stroke using the MOD-HOSTILE score.

With increasing threshold probabilities, the net benefit associated with the MOD-HOSTILE score progressively declined. At threshold probabilities around 20%, the MOD-HOSTILE curve approached the zero net benefit line and subsequently ran nearly parallel to the baseline, indicating no additional net benefit at higher thresholds. Throughout, the MOD-HOSTILE curve remained consistently below the treat-all strategy, reflecting that universal treatment would yield a higher net benefit at these higher thresholds. Figure 8 illustrates the decision curve analysis, highlighting both the intersection with the treat-none strategy and the relative position below the treat-all curve.

### 3.2. Femoral Cannulation

Patients in the high MOD-HOSTILE group were significantly older (median 70.00 [61.50–75.00] years vs. 61.00 [53.75–68.00] in the low group; *p* < 0.01) and had more comorbidities (Table 2), including higher EuroSCORE II (1.70 [1.19–2.12] vs. 0.82 [0.61–1.25]; *p* < 0.01), arterial hypertension (91.67% vs. 48.82%; *p* < 0.01), and diabetes mellitus (25% vs. 5.21%; *p* = 0.02). BMI also increased with each MOD-HOSTILE category (26.85 [22.70–32.50] vs. 24.91 [22.87–28.00]; *p* = 0.09).

Although stroke rates in femoral-cannulated patients tended to be higher in the high MOD-HOSTILE group (0% in high vs. 0.95% [2/211] in low; *p* = 0.64), the difference was not statistically significant. Delirium and other neurological events were more frequent in the high MOD-HOSTILE group, though not statistically significant.

High MOD-HOSTILE patients were associated with significantly more postoperative complications, including pRBC transfusions (median 2.00 [0.00–3.00] vs. 0.00 [0.00–2.00]; *p* < 0.01), prolonged ICU stay (2.00 [1.00–3.00] vs. 2.00 [1.00–2.00]; *p* = 0.02), and prolonged ventilation >24 h (16.67% vs. 4.27%; *p* = 0.02). Hospital stay was slightly longer in the high MOD-HOSTILE category (11.00 [7.00–15.00] vs. 10.00 [8.00–12.00]; *p* < 0.01).

Thirty-day and in-hospital mortality did not differ significantly between MOD-HOSTILE categories (0% vs. 0.95%; *p* = 0.51) (Table 2).

### 3.3. Axillary Cannulation

Axillary cannulation was used only when femoral access was contraindicated. Patients in the high MOD-HOSTILE group had a higher baseline MOD-HOSTILE score (median 6.00 [5.00–8.00]) compared to the low (3.00 [2.00–4.00]) and mild (4.00 [3.00–5.00]) groups. Stroke occurred in 18.18% (2/11) of patients in the high MOD-HOSTILE group versus 0% in the low and mild groups (*p* = 0.04).

Temporary neurological complications occurred in 9.09% of high MOD-HOSTILE patients compared to 0–18.75% in the low and mild groups (*p* = 0.16). ICU stay (median 2.00 [1.00–6.00] days) and prolonged ICU stay (>3 days, 33.33%) were higher in the high MOD-HOSTILE group, though not statistically significant. Hospital stay (11.00 [8.00–16.00] days) was slightly longer than in the low and mild groups (9.50 [7.50–12.00] and 9.00 [7.00–12.00] days, *p* = 0.47). Thirty-day mortality was 9.09% (1/11) in the high MOD-HOSTILE group versus 0–6.25% in the low and mild groups (*p* = 0.31) (Table 3).

## 4. Discussion

This study underscores the importance of thorough preoperative vascular assessment in minimally invasive heart valve surgery employing peripheral cannulation. Patients with higher MOD-HOSTILE scores demonstrated a substantially increased risk of neurological complications. In the overall cohort, stroke occurred more frequently with increasing MOD-HOSTILE category and was most pronounced in the high-risk group (8.7%), despite careful access planning and the use of alternative cannulation strategies. These findings indicate that advanced peripheral vascular disease confers a persistent embolic risk that is not fully attenuated by changing the cannulation site.

The observed increase in stroke incidence across rising MOD-HOSTILE categories, with the highest rate observed in the high-risk group (8.70%, *p* = 0.02), confirms the score’s ability to identify patients with a substantially elevated neurological risk. These findings indicate that the MOD-HOSTILE score effectively stratifies patients according to stroke risk and retains prognostic relevance, particularly at the upper end of the risk spectrum.

The observed increase in stroke incidence across rising MOD-HOSTILE categories, with the highest event rate in the high-risk group (8.70%, *p* = 0.02), confirms the score’s ability to identify patients with a substantially elevated neurological risk. This graded association supports the prognostic validity of the MOD-HOSTILE score and is consistent with its intended role as a risk stratification tool.

DCA complements these findings by shifting the focus from discrimination to clinical decision-making. As originally described by Vickers and Elkin, DCA evaluates the net clinical benefit of prediction models across a range of threshold probabilities and does not require a model to outperform a treat-all strategy at all thresholds to be considered clinically useful [11]. Importantly, for severe outcomes such as stroke, low threshold probabilities are often clinically relevant, reflecting a low tolerance for missed events [12].

In this context, the MOD-HOSTILE score demonstrates its greatest clinical value at lower threshold probabilities, where it provides a higher net benefit than both treat-all and treat-none strategies. This finding indicates that the score supports early risk stratification and decision-making in scenarios where clinicians are willing to intervene at relatively low estimated stroke risks.

At higher threshold probabilities, the net benefit of the MOD-HOSTILE score converges with the treat-none strategy and remains below treat-all. This pattern does not indicate poor model performance but rather reflects a well-recognized phenomenon in decision curve analysis of stroke prediction models. Previous studies have shown that for high-impact neurological outcomes, treat-all strategies may dominate at higher thresholds because patients exceeding these thresholds already represent an evidently high-risk population, limiting the incremental decision value of additional risk stratification [11].

While high MOD-HOSTILE scores remain prognostically meaningful and identify patients at markedly increased stroke risk, their added value for decision-making at higher thresholds is inherently limited. Overall, the decision curve analysis supports the MOD-HOSTILE score as a clinically useful tool primarily for early risk identification, rather than for guiding decisions at high risk thresholds where treatment decisions are typically evident.

In the axillary cannulation subgroup, patients exhibited significantly higher baseline MOD-HOSTILE scores, reflecting a greater vascular disease burden. Notably, stroke occurred exclusively in patients within the high MOD-HOSTILE category (18.18%), whereas no strokes were observed in the low or mild categories. Although patient numbers were limited, this finding suggests that axillary cannulation does not eliminate neurological risk in patients with advanced systemic atherosclerosis. Similarly, in the femoral cannulation cohort, higher MOD-HOSTILE scores were associated with increased rates of postoperative delirium and other neurological complications, supporting the hypothesis that retrograde perfusion in the presence of hostile vascular anatomy contributes to cerebral embolization.

Patients in the high MOD-HOSTILE category exhibited a significantly higher burden of comorbidities, including arterial hypertension, diabetes mellitus, peripheral artery disease, and impaired renal function, consistent with a more advanced systemic atherosclerotic profile. Accordingly, postoperative acute renal injury and the need for renal replacement therapy occurred more frequently in higher-risk groups, emphasizing the need for intensified perioperative renal protection strategies in this population. In addition, higher MOD-HOSTILE scores were associated with increased postoperative morbidity, including repeat intubation, prolonged mechanical ventilation, extended ICU stay, and infectious complications such as pneumonia and sepsis. These findings highlight not only the increased procedural risk but also the greater resource utilization associated with advanced vascular disease.

Procedure type differed significantly across MOD-HOSTILE categories. Aortic valve procedures were more frequently performed in patients with high MOD-HOSTILE scores, whereas mitral valve procedures predominated in the low-risk group. This observation may reflect the close association between aortic valve pathology and generalized vascular calcification, which may further amplify embolic risk during retrograde perfusion and peripheral cannulation.

Our findings align with prior work by Singh et al. who demonstrated that the original HOSTILE score effectively identifies patients with challenging iliofemoral anatomy undergoing transfemoral TAVR, while still allowing successful treatment in the majority of cases [1,2,11,12]. Similarly, our data show that minimally invasive valve surgery remains feasible in patients with elevated MOD-HOSTILE scores; however, these patients experience significantly higher rates of neurological and systemic complications. This underscores the need for enhanced risk stratification and tailored perioperative management strategies rather than simple exclusion based on vascular anatomy.

Traditional surgical risk models, such as the STS score, do not adequately capture vascular-specific risks relevant to minimally invasive cardiac surgery. While the STS score was developed and validated for conventional cardiac surgery and focuses predominantly on cardiac and systemic risk factors, it does not account for peripheral vessel diameter, calcification, or tortuosity, key determinants of cannulation safety and retrograde perfusion dynamics [13]. In contrast, the MOD-HOSTILE score directly addresses these vascular characteristics and may serve as a surrogate marker of global atherosclerotic burden. Ascending aortic and arch pathology are not included in the score, as such patients are typically excluded from minimally invasive approaches during preoperative imaging assessment.

Importantly, our analysis suggests a clinically relevant threshold at a MOD-HOSTILE score of ≥5 points, beyond which the risk of adverse neurological and systemic outcomes increases markedly. While this threshold appears meaningful within our cohort, it requires further validation in larger, multicenter studies before widespread clinical adoption.

## 5. Conclusions

We introduce the MOD-HOSTILE score as a tool for risk stratification in minimally invasive heart valve surgery. Higher MOD-HOSTILE scores were associated with an increased risk of stroke, and in patients undergoing femoral cannulation, also with a higher risk of postoperative delirium. A score of ≥5 points identified patients at particularly elevated neurological and systemic risk, suggesting that alternative surgical strategies or enhanced perioperative planning may be warranted for this group.

Our findings underscore the importance of preoperative vascular assessment, as patients with advanced peripheral vascular disease remain at risk for embolic and systemic complications despite careful access planning. Decision curve analysis indicates that the MOD-HOSTILE score provides the greatest clinical benefit at lower intervention thresholds, supporting early risk identification and management. At higher thresholds, while high scores remain prognostically informative, their incremental decision-making value is limited.

Overall, the MOD-HOSTILE score offers a practical, anatomy-focused complement to traditional surgical risk models such as the STS score, enabling more tailored perioperative strategies and improved risk awareness in patients undergoing minimally invasive cardiac surgery.

## Figures and Tables

**Table 1:** MOD-HOSTILE score—results from the overall cohort.

Overall Results	Low MOD-HOSTILE Category (n = 228)	Mild MOD-HOSTILE Category (n = 113)	High MOD-HOSTILE Category (n = 23)	*p*-Value
**Preoperative results**				
**Age, IQR**	61.00 [60.00–63.00]	68.00 [66.60–70.00]	70.00 [64.70–73.00]	<0.01
Male, n (%)	136 (59.65)	69 (61.06)	16 (69.57)	0.65
BMI (kg/m^2^), IQR	24.90 [24.40–25.50]	25.65 [24.90–27.03]	24.70 [22.40–30.12]	0.10
BSA (m^2^), SD	1.92 (1.89–1.95)	1.95 (1.90–1.99)	1.92 (1.89–1.95)	0.56
**Arterial hypertension n (%)**	**114 (50)**	**78 (69.03)**	**17 (73.91)**	**<0.01**
Atrial fibrillation, n (%)	36 (15.79)	26 (23.01)	4 (17.39)	0.26
History of stroke, n (%)	5 (2.19)	4 (3.54)	2 (8.70)	0.29
**COPD, n (%)**	**7 (3.07)**	**4 (3.54)**	**4 (17.39)**	**<0.01**
**History of smoking, n (%)**	**37 (16.23)**	**32 (28.32)**	**13 (56.52)**	**<0.01**
Creatinine level, SD	0.95 ±0.69	0.94 ±0.42	0.99 ±1.03	0.13
**eGFR (mL/min/1.73 m^2^), IQR**	88.17 [83.63–90.00]	81.98 [78.00–85.23]	75.57 [68.36–84.34]	<0.01
Diabetes mellitus, n (%)	13 (5.70)	12 (10.62)	4 (17.39)	0.06
**Carotid stenosis > 50% unilateral**	**0**	**1 (0.88)**	**1 (4.35)**	**0.02**
**PAD, n (%)**	**2 (0.88)**	**5 (4.42)**	**2 (8.70)**	**0.02**
**Operated on PAD, n (%)**	**1 (0.44)**	**0**	**1 (4.35)**	**0.03**
**Previous PCI, n (%)**	**98 (42.98)**	**65 (57.52)**	**4 (17.39)**	**<0.01**
**NYHA_classification, n (%)**				**<0.01**
- **I**	**64 (28.07)**	**20 (17.70)**	**1 (4.35)**	
- **II**	**114 (50)**	**47 (41.49)**	**6 (26.09)**	
- **III**	**44 (19.30)**	**39 (34.51)**	**13 (56.52)**	
- **IV**	**5 (2.19)**	**6 (5.31)**	**3 (13.04)**	
**EuroSCORE II, IQR**	**0.83 [0.75–0.94]**	**1.04 [0.91–1.22]**	**1.79 [1.26–2.16]**	**<0.01**
LVEF (%), IQR	58.35 [56.20–60.00]	57.65 [56.00–60.00]	58.50 [52.91–65.00]	0.49
**Intraoperative results**				
**MV repair/replacement, n (%)**	**165 (72.37)**	**101 (89.38)**	**11 (47.83)**	**<0.01**
**AV replacement, n (%)**	**47 (20.61)**	**12 (10.62)**	**10 (43.48)**	
**TV repair, n (%)**	**3 (1.32)**	**0**	**1 (4.35)**	
**MV repair/replacement + TV repair, n (%)**	**9 (3.95)**	**0**	**0**	
**MV repair/replacement + AV replacement, n (%)**	**4 (1.75)**	**0**	**1 (4.35)**	
**Cannulation, n (%)** - **Axillary cannulation** - **Femoral cannulation**	**16 (7.02)** **212 (92.98)**	**18 (15.93)** **95 (84.07)**	**11 (47.83)** **12 (52.17)**	**<0.01**
Cross-clamp time (min.), SD	84.82 ± 31.72	78.82 ± 41.69	79.17 ± 126.94	0.22
Intraoperative NIRS drop ≥ 20% during CPB, n (%)	2 (0.88)	4 (3.54)	1 (4.35)	0.37
**Postoperative results**				
Delirium, n (%)	14 (6.14)	14 (12.39)	3 (13.04)	0.29
**Stroke, n (%)**	**2 (0.88)**	**2 (1.77)**	**2 (8.70)**	**0.02**
Seizure, n (%)	6 (2.63)	2 (1.77)	1 (4.35)	0.74
TIA, n (%)	0	1 (0.88)	0	0.33
Aphasia, n (%)	0	1 (0.88)	0	0.32
**Hemiplegia, n (%)**	**2 (0.88)**	**2 (1.77)**	**3 (13.04)**	**<0.01**
Paraplegia, n (%)	0	1 (0.88)	0	0.33
Coma, n (%)	0	2 (1.77)	0	0.34
Temporary neurological complications *, n (%)	**4 (1.75)**	**0**	**2 (8.67)**	**0.01**
Combined endpoint (Stroke, Seizure, TIA, neurological symptoms, malperfusion, Delirium, delayed awakening), n (%)	25 (10.69)	20 (17.70)	5 (21.74)	0.12
**Combined endpoint (monoplegia, paraplegia, hemiplegia, cerebral malperfusion, upper and lower extremity malperfusion), n (%)**	**6 (2.63)**	**7 (6.19)**	**5 (21.74)**	**<0.01**
Lower extremity malperfusion, n (%)	3	1	0	0.94
Deep wound infection, n (%)	2 (0.88)	0	1 (4.35)	0.11
Acute renal failure, n (%)	8 (3.51)	11 (9.73)	1 (4.35)	0.06
Acute renal failure requiring dialysis, n (%)	4 (1.75)	6 (5.31)	1 (4.35)	0.18
FFP’s, IQR	0.00 [0.00–0.00]	0.00 [0.00–0.00]	0.00 [0.00–0.65]	0.13
**pRBC’s, IQR**	**0.00 [0.00–0.00]**	**0.50 [0.00–1.00]**	**2.00 [0.00–2.65]**	**0.01**
**Thrombocytes, SD**	**0.00 [0.00–0.00]**	**0.00 [0.00–0.00]**	**0.00 [0.00–0.00]**	**0.03**
Pneumonia, n (%)	7 (3.07)	9 (7.96)	1 (4.35)	0.13
**Repeated intubation, n (%)**	**6 (2.63)**	**15 (13.27)**	**3 (13.04)**	**<0.01**
**Prolonged ventilation (>24 h), n (%)**	**11 (4.82)**	**12 (10.62)**	**4 (17.39)**	**0.03**
ICU stay (days), IQR	2.00 [1.00–2.00]	2.00 [1.00–2.00]	2.00 [1.00–3.00]	0.18
Prolonged ICU stay (<3 days), n (%)	28 (12.28)	28 (24.78)	5 (21.74)	0.11
Sepsis, n (%)	1 (0.44)	5 (4.42)	0	0.06
ECMO, n (%)	4 (1.75)	4 (3.54)	1 (4.35)	0.75
Hospital stay (days), IQR	10.00 [9.00–10.00]	11.50 [10.00–13.85]	11.00 [7.00–13.65]	<0.01
30-day mortality rate, n (%)	3 (1.32)	2 (1.77)	0	0.09

SD: standard deviation, n: number, BMI: body mass index, BSA: body surface area, COPD: chronic obstructive pulmonary disease, eGFR: estimated glomerular filtration rate, PAD: peripheral arterial disease, PCI: percutaneous coronary intervention, NYHA: New York Heart Association classification, LVEF: left ventricular ejection fraction, MV: mitral valve, AV: aortic valve, TV: tricuspid valve, NIRS: near-infrared spectroscopy, CPB: cardiopulmonary bypass, TIA: transient ischemic attack, FFP: fresh frozen plasma, pRBC: packed red blood cells, ICU: intensive care unit, ECMO: extracorporeal membrane oxygenation, IQR: interquartile range. * Temporary neurological complications are transient, self-limiting neurological symptoms that resolve without permanent deficits. These may include visual disturbances such as eye flickering or seeing flashing lights, as well as sensory symptoms like paresthesia in the fingers, hands, or feet.

**Table 2:** MOD-HOSTILE score—Evaluation for patients cannulated at the femoral artery.

	Low MOD-HOSTILE Category (n = 211)	Mild MOD-HOSTILE Category (n = 92)	High MOD-HOSTILE Category (n = 12)	*p*-Value	
**Preoperative results**					
**Age at surgery, CI**	**61.00 [53.75–68.00]**	**68.00 [60.00–73.00]**	**70.00 [61.50–75.00]**	**<0.01**	
Male, n (%)	127 (60.19)	60 (65.22)	9 (75)	0.53	
BMI (kg/m^2^), IQR	24.91 [22.87–28.00]	25.95 [23.72–29.71]	26.85 [22.70–32.50]	0.09	
BSA (m^2^), SD	1.92 ± 0.18	1.96 ± 0.37	1.96 ± 1.33	0.36	
**Arterial hypertension, n (%)**	**103 (48.82)**	**68 (73.91)**	**11 (91.67)**	**<0.01**	
History of stroke, n (%)	5 (2.37)	4 (4.35)	1 (8.33)	0.39	
COPD, n (%)	6 (2.84)	3 (3.26)	1 (8.33)	0.57	
History of smoking, n (%)	54 (25.59)	22 (23.91)	6 (50)	0.26	
Creatinine level, IQR	0.85 [0.70–1.01]	0.91 [0.79–1.03]	0.93 [0.84–1.06]	0.08	
**eGFR** **(mL/min/1.73 m^2^), IQR**	**88.90 [71.41–98.22]**	**81.10 [69.00–91.60]**	**78.31 [64.08–87.45]**	**<0.01**	
**Diabetes mellitus, n (%)**	**11 (5.21)**	**9 (9.78)**	**3 (25)**	**0.02**	
Carotis stenosis > 50%, unilateral, n (%)	0	1 (1.09)	0	0.30	
**Peripheral artery disease (PAD), n (%)**	**2 (0.95)**	**5 (5.43)**	**1 (8.33)**	**0.03**	
**Previous PCI, n (%)**	**93 (44.08)**	**59 (64.13)**	**2 (16.67)**	**<0.01**	
**NYHA_classification, n (%)**				**<0.01**	
- **I**	**61 (28.91)**	**17 (18.48)**	**0**		
- **II**	**107 (50.71)**	**40 (43.48)**	**3 (25)**		
- **III**	**41 (19.43)**	**34 (36.96)**	**7 (58.33)**		
- **IV**	**3 (1.42)**	**3 (3.26)**	**2 (16.67)**		
**Euroscore II, IQR**	**0.82 [0.61–1.25]**	**0.98 [0.69–1.46]**	**1.70 [1.19–2.12]**	**<0.01**	
LVEF (%), IQR	58.00 [55.00–65.00]	57.65 [55.00–63.00]	57.95 [48.00–65.00]	0.66	
**Intraoperative results**					
**MV repair/replacement n (%)**	**156 (73.93)**	**83 (90.22)**	**5 (41.67)**	**<0.01**	
**AV replacement n (%)**	**42 (19.91)**	**12 (13.04)**	**7 (58.22)**		
**TV repair n (%)**	**3 (1.42)**	**0**	**0**		
**MV procedure + TV repair n (%)**	**8 (3.79)**	**0**	**0**		
**MV procedure + AV replacement n (%)**	**3 (1.42)**	**0**	**0**		
Crossclamping time (min.), IQR	75.00 [65.00–95.25]	75.00 [61.00–93.00]	86.50 [66.50–114.00]	0.57	
Intraoperative NIRS drop ≥ 20% during CPB, n (%)	2 (0.95)	4 (4.35)	0	0.34	
Extubated in the OR, n (%)	152 (72.04)	61 (66.30)	9 (75)	0.73	
**Postoperative results**					
Delirium, n (%)	13 (6.16)	13 (14.13)	2 (16.67)	0.06	
Stroke, n (%)	2 (0.95)	2 (2.17)	0	0.64	
Seizure, n (%)	5 (2.37)	2 (2.17)	1 (8.33)	0.70	
TIA, n (%)	0	0	0	-	
Aphasia, n (%)	0	1 (1.09)	0	0.30	
**Hemiplegia, n (%)**	**2 (0.95)**	**0**	**1 (8.33)**	**0.04**	
Paraplegia, n (%)	0	1 (1.09)	0	0.30	
Coma, n (%)	0	2 (2.17)	0	0.31	
Temporary neurological complication *, n (%)	**1 (0.47)**	**0**	**1 (8.33)**	**<0.01**	
Combined endpoint (Stroke, Seizure, TIA, neurological symptoms, malperfusion, Delirium, delayed awakening), n (%)	22 (10.42)	17 (18.48)	2 (16.67)	0.17	
Combined endpoint (monoplegia, paraplegia, hemiplegia, cerebral malperfusion, upper and lower extremity malperfusion), n (%)	4 (1.90)	6 (6.52)	1 (8.33)	0.09	
Lower extremity malperfusion, n (%)	2	1	0	0.94	
**Deep wound infection, n (%)**	**2 (0.95)**	**0**	**1 (8.33)**	**0.02**	
**Acute renal injury, n (%)**	**8 (3.79)**	**11 (11.96)**	**1 (8.33)**	**0.03**	
Acute renal injury requiring dialysis, n (%)	4 (1.90)	6 (6.52)	1 (8.33)	0.09	
FFPs, SD	0.00 [0.00–0.00]	0.00 [0.00–0.00]	0.00 [0.00–1.00]	0.31	
**pRBC, SD**	**0.00 [0.00–2.00]**	**1.00 [0.00–4.00]**	**2.00 [0.00–3.00]**	**<0.01**	
Thrombocytes number, SD	0.27 ± 1.25	0.49 ± 1.77	0.83 ± 6.15	0.18	
**Pneumonia, n (%)**	**6 (2.85)**	**9 (9.78)**	**1 (8.33)**	**0.04**	
**Repeat intubation, n (%)**	**6 (2.85)**	**15 (16.30)**	**1 (8.33)**	**<0.01**	
**Prolonged ventilation (>24 h), n (%)**	**9 (4.27)**	**11 (11.96)**	**2 (16.67)**	**0.02**	
**ICU stay (days), SD**	**2.00 [1.00–2.00]**	**2.00 [1.00–4.00]**	**2.00 [1.00–3.00]**	**0.02**	
**Prolonged ICU stay (>3 days), n (%)**	**25 (11.85)**	**28 (30.43)**	**2 (16.67)**	**0.01**	
**Sepsis, n (%)**	**0**	**4 (4.35)**	**0**	**0.03**	
ECMO, n (%)	3 (1.42)	4 (4.43)	1 (8.33)	0.37	
**Hospital stay (days), IQR**	**10.00 [8.00–12.00]**	**12.50 [9.00–18.00]**	**11.00 [7.00–15.00]**	**<0.01**	
30-day mortality, n (%)	2 (0.95)	3 (3.26)	0	0.51	

SD: standard deviation, n: number, BMI: body mass index, BSA: body surface area, COPD: chronic obstructive pulmonary disease, eGFR: estimated glomerular filtration rate, PAD: peripheral arterial disease, PCI: percutaneous coronary intervention, NYHA: New York Heart Association classification, LVEF: left ventricular ejection fraction, MV: mitral valve, AV: aortic valve, TV: tricuspid valve, NIRS: near-infrared spectroscopy, CPB: cardiopulmonary bypass, TIA: transient ischemic attack, FFP: fresh frozen plasma, pRBC: packed red blood cells, ICU: intensive care unit, ECMO: extracorporeal membrane oxygenation, IQR: interquartile range. * Temporary neurological complications are transient, self-limiting neurological symptoms that resolve without permanent deficits. These may include visual disturbances such as eye flickering or seeing flashing lights, as well as sensory symptoms like paresthesias in the fingers, hands, or feet.

**Table 3:** MOD-HOSTILE score—Evaluation for patients cannulated at the axillary artery.

	Low MOD-HOSTILE Category (n = 16)	Mild MOD-HOSTILE Category (n = 18)	High MOD-HOSTILE Category (n = 11)	*p*-Value
**Preoperative results**				
**Age, IQR**	63.50 [59.00–70.50]	70.00 [67.00–74.00]	69.00 [66.50–73.00]	0.049
Male, n (%)	9 (56.25)	9 (50)	7 (63.64)	0.77
BMI (kg/m^2^), SD	24.74 [22.00–28.70]	24.90 [23.00–30.50]	24.10 [21.42–29.15]	0.55
BSA (m^2^), SD	1.90 ± 0.22	1.87 ± 0.23	1.84 ± 0.26	0.78
Arterial hypertension, n (%)	11 (68.75)	11 (61.11)	6 (54.55)	0.75
History of stroke, n (%)	0	0	1 (9.09)	0.21
COPD, n (%)	1 (6.25)	1 (5.56)	3 (27.27)	0.15
**History of smoking**, n (%)	3 (18.75)	10 (55.56)	7 (63.64)	0.02
eGFR (mL/min/1.73 m^2^), SD	79.75 [62.28–85.44]	85.12 [70.95–92.70]	74.29 [66.80–80.07]	0.42
Diabetes_Mellitus, n (%)	2 (12.50)	3 (16.67)	1 (9.09)	0.84
Carotid stenosis > 50% unilateral, n (%)	0	0	1 (9.09)	0.21
PAD, n (%)	0	0	1 (9.09)	0.21
NYHA_classification, n (%)				0.67
- I	4 (25)	3 (16.67)	1 (9.09)	
- II	7 (43.75)	7 (38.89)	3 (27.27)	
- III	3 (18.75)	5 (27.78)	6 (54.55)	
- IV	2 (12.50)	3 (16.67)	1 (9.09)	
Euroscore II, SD	1.10 [0.75–2.39]	1.32 [0.95–2.44]	1.90 [1.11–4.99]	0.13
LVEF (%), SD	59.90 [55.00–67.10]	57.65 [53.00–65.00]	57.70 [51.50–63.75]	0.61
**Intraoperative results**				
MV repair/replacement n (%)	9 (56.25)	18 (100)	6 (54.55)	0.07
AV replacement n (%)	5 (31.25)	0	3 (27.27)	
TV repair n (%)	0	0	1 (9.09)	
MV repair/replacement + TV repair n (%)	1 (6.25)	0	0	
AV replacement + MV repair/replacement, n (%)	1 (6.25)	0	1 (9.09)	
Cross-clamping time (min.), SD	80.50 [57.00–116.50]	59.50 [50.00–80.00]	58.00 [40.00–80.75]	0.11
Intraoperative NIRS drop ≥20% during CPB, n (%)	**0**	**0**	**1 (9.09)**	**0.21**
**Postoperative results**				
Delirium, n (%)	2 (12.50)	2 (11.11)	1 (9.09)	0.74
**Stroke, n (%)**	**0**	**0**	**2 (18.18)**	**0.04**
Seizure, n (%)	0	1 (5.56)	0	0.74
TIA, n (%)	0	1 (5.56)	0	0.67
Aphasia, n (%)	0	0	0	-
Hemiplegia, n (%)	0	1 (5.56)	2 (18.18)	0.17
Paraplegia, n (%)	0	0	0	-
Coma, n (%)	0	0	0	-
Temporary neurological complications *, n (%)	3 (18.75)	0	1 (9.09)	0.16
Combined endpoint (Stroke, Seizure, TIA, neurological symptoms, malperfusion, Delirium, delayed awakening), n (%)	3 (18.75)	3 (16.67)	3 (27.27)	0.78
Combined endpoint (monoplegia, paraplegia, hemiplegia, cerebral malperfusion, upper and lower extremity malperfusion), n (%)	2 (12.50)	1 (5.56)	4 (36.36)	0.07
Malperfusion of lower extremities, n (%)	1	0	0	0.40
Deep wound infection, n (%)	0	0	0	-
Acute renal failure, n (%)	0	0	0	-
Acute renal failure requiring dialysis, n (%)	0	0	0	-
Pneumonia, n (%)	1 ± 6.25	0	0	0.60
**Repeat intubation, n (%)**	**0**	**0**	**2 (18.18)**	**0.04**
ICU stay (days), SD	2.00 [1.00–3.50]	1.00 [1.00–2.00]	2.00 [1.00–6.00]	0.19
Prolonged ICU stay (>3 days)	4 (25)	1 (5.56)	3 (33.33)	0.23
Sepsis, n (%)	0	0	0	-
ECMO, n (%)	1 (6.25)	0	0	0.60
Hospital stay (days), SD	9.50 [7.50–12.00]	9.00 [7.00–12.00]	11.00 [8.00–16.00]	0.47
30-day mortality, n (%)	1 (6.25)	0	1 (9.09)	0.31

SD: standard deviation, n: number, BMI: body mass index, BSA: body surface area, COPD: chronic obstructive pulmonary disease, eGFR: estimated glomerular filtration rate, PAD: peripheral arterial disease, PCI: percutaneous coronary intervention, NYHA: New York Heart Association classification, LVEF: left ventricular ejection fraction, MV: mitral valve, AV: aortic valve, TV: tricuspid valve, NIRS: near-infrared spectroscopy, CPB: cardiopulmonary bypass, TIA: transient ischemic attack, ICU: intensive care unit, ECMO: extracorporeal membrane oxygenation, IQR: interquartile range. * Temporary neurological complications are transient, self-limiting neurological symptoms that resolve without permanent deficits. These may include visual disturbances such as eye flickering or seeing flashing lights, as well as sensory symptoms like paresthesia in the fingers, hands, or feet.

## Data Availability

Due to ethical and privacy considerations, some data may be restricted, but can be made available to qualified researchers upon formal request.

## Abbreviations

The following abbreviations are used in this manuscript:

BMI	body mass index
BSA	body surface area
LVEF	Left ventricular ejection fraction
PAD	peripheral arterial disease
COPD	chronic obstructive pulmonary disease
TIA	transient ischemic attack
pRBC	packed red blood cells
ICU	intensive care unit
FFP	fresh frozen plasma
GI	bleeding gastrointestinal bleeding
MAE	major adverse event
TAVR	transcatheter aortic valve replacement
MOD-HOSTILE	modified HOSTILE score
PCI	percutaneous coronary intervention
NYHA	New York heath association
HU	Hounsfield units
eGFR	estimated glomerular filtration rate
MV	mitral valve
MIVS	Minimally invasive heart valve surgery
AV	aortic valve
TV	tricuspid valve
NIRS	near-infrared spectroscopy
CPB	cardiopulmonary bypass
ECMO	extracorporeal membrane oxygenation
GCS	Glasgow coma scale
CAM-ICU	Confusion Assessment Method for the Intensive Care Unit
RAMT	right anterolateral mini-thoracotomy
ECG	electrocardiogram
Pt	threshold propability
DCA	decision curve analysis
IQR	interquartile range
SD	standard deviation
